# Men's Preferences for Women's Femininity in Dynamic Cross-Modal Stimuli

**DOI:** 10.1371/journal.pone.0069531

**Published:** 2013-07-31

**Authors:** Jillian J. M. O'Connor, Paul J. Fraccaro, Katarzyna Pisanski, Cara C. Tigue, David R. Feinberg

**Affiliations:** Department of Psychology, Neuroscience & Behaviour, McMaster University, Hamilton, Ontario, Canada; University of Nottingham Malaysia Campus, Malaysia

## Abstract

Men generally prefer feminine women's faces and voices over masculine women's faces and voices, and these cross-modal preferences are positively correlated. Men's preferences for female facial and vocal femininity have typically been investigated independently by presenting soundless still images separately from audio-only vocal recordings. For the first time ever, we presented men with short video clips in which dynamic faces and voices were simultaneously manipulated in femininity/masculinity. Men preferred feminine men's faces over masculine men's faces, and preferred masculine men's voices over feminine men's voices. We found that men preferred feminine women's faces and voices over masculine women's faces and voices. Men's attractiveness ratings of both feminine and masculine faces were increased by the addition of vocal femininity. Also, men's attractiveness ratings of feminine and masculine voices were increased by the addition of facial femininity present in the video. Men's preferences for vocal and facial femininity were significantly and positively correlated when stimuli were female, but not when they were male. Our findings complement other evidence for cross-modal femininity preferences among male raters, and show that preferences observed in studies using still images and/or independently presented vocal stimuli are also observed when dynamic faces and voices are displayed simultaneously in video format.

## Introduction

Mating effort is a finite resource [1], as time and energy allocated to finding potential mates cannot be used for other activities. One way in which organisms may increase the efficiency and efficacy of mate-search behaviour is by assessing potential mates on multiple cues to underlying quality [2], [3]. In humans, men's mate preferences are influenced by both vocal and facial femininity. Men prefer feminine women's faces over masculine women's faces [4], [5], [6], [7]. Men also prefer higher pitched women's voices over lower pitched women's voices [8], [9], [10], [11], [12]. Men's preferences for vocal and facial femininity in women are positively correlated [5], suggesting that men assess feminine female traits similarly across modalities.

Women with more feminine faces also have more feminine voices [13]. Women with more feminine- and attractive-looking faces also have higher levels of estrogen during the late follicular phase of the menstrual cycle, which is the time when conception risk is highest [14]. Furthermore, women who have relatively more feminine faces, as measured objectively via facial metrics and subjectively via ratings, also have higher pitched voices [13], [15]. Although both facial and vocal femininity predict women's attractiveness to men [4], [5], [6], [7], [8], [9], [10], [11], [12], a recent study failed to find a significant relationship between women's estrogen levels and participants' perceptions of their facial or vocal attractiveness [16]. However, this study did not include an analysis of the relationship between women's estrogen levels and their facial femininity or their voice pitch. Moreover, many other traits besides femininity contribute to perceptions of attractiveness. For example, skin coloration [17], [18], apparent health [19], [20], and symmetry [21], [22] also contribute to women's facial attractiveness whereas formant frequencies [11], [12], [23], articulation [24], and nasality [24] further contribute to women's vocal attractiveness. Thus, these additional factors may have masked the relationship between women's estrogen levels and their vocal and facial attractiveness. Therefore, while previous research has demonstrated a positive relationship between women's estrogen levels and facial femininity [14], and between facial femininity and vocal femininity [13], [15], the evidence for a direct relationship between women's vocal and facial attractiveness and women's estrogen levels is equivocal.

By preferentially mating with relatively feminine women, men may gain fitness benefits stemming from fertility, reproductive value, and health. Women with higher levels of trait estrogen express a desire for higher numbers of offspring than do women with lower levels of trait estrogen [25]. Such women are also more likely to be able to conceive offspring, given that estrogen levels are positively associated with the probability of conception [26]. Therefore, men who prefer relatively feminine women may benefit from increased reproductive success.

Younger women's voices are perceived as both more attractive and more feminine than are the voices of peri- and post-menopausal women [27]. Women with higher pitched voices are also perceived as younger than are women with lower pitched voices [8], [28]. Women's voice pitch decreases with increasing age [28], [29] as do indices of fertility such as oocyte quality and quantity [30]. Age-related changes in women's voice pitch may be due in part to a decrease in estrogen production relative to testosterone that occurs during menopause [28], which in turn increases the mass of the vocal folds [31]. Larger vocal folds vibrate at a slower rate than do smaller vocal folds, resulting in lower fundamental frequency, the acoustic correlate of voice pitch [31]. Among post-pubertal women, younger women are more likely to conceive [32], and have greater reproductive potential [30] than do older women. These findings further evidence that vocal femininity may be preferred by men on the basis of positive associations with fertility.

Men may also prefer feminine characteristics in potential mates due to positive associations with indices of health. Feminine women's faces are perceived as healthier than are masculine women's faces [7]. Women with relatively more feminine faces also report significantly fewer respiratory illnesses [7], [33] and less antibiotic use [7] than do women with relatively more masculine faces. However, female facial femininity is not always positively associated with aspects of health. For example, women's facial femininity is not significantly associated with stomach/intestinal infections [7], [33]. Nonetheless, research on men's preferences for women's faces suggests that health is one of the potential fitness benefits of men's preferences for female femininity. For instance, men's scores on a scale of pathogen disgust, but not moral or sexual disgust, are positively associated with their preferences for feminine women's faces [34]. Furthermore, priming men to the presence of contagious pathogens results in stronger preferences for female facial femininity in comparison to preferences reported before priming [35]. If feminine women are healthier than are relatively masculine women, then offspring may benefit by increased provisioning and/or heritable health.

Research on men's face preferences using dynamic stimuli has typically focused on the attractiveness of faces across dynamic and still images. For example, Morrison and colleagues presented viewers with faces that were manipulated to be more or less feminine and found significant preferences for femininity in both static photographs and in soundless recordings of moving women's faces [36]. Masculinity manipulations, however, did not influence preferences for moving or static male faces. Also, Lander compared the attractiveness of still and dynamic faces, with the addition of attractiveness ratings of separately presented voices [37]. Here, Lander presented men with unmanipulated men's and women's voices separately from either unmanipulated soundless still or dynamic images of women's and men's faces. Lander found that men's attractiveness ratings of both still and dynamic women's faces and voices were significantly positively correlated, although men's attractiveness ratings of other men's faces and voices were not, regardless of whether faces were still or dynamic.

Despite the growing body of research on facial and vocal attractiveness using video stimuli, research to date has only tested men's preferences for female facial and vocal femininity by presenting such stimuli separately and by using static stimuli. For example, Fraccaro and colleagues presented men with still images of women's faces separately from audio-only recordings of women's voices [5]. The authors found significant preferences among men for feminized women's voices and faces over masculinized women's voices and faces. Furthermore, men who preferred feminized women's voices also preferred feminized women's faces. It is unclear, however, whether men's preferences for female femininity are observed when faces and voices are presented simultaneously.

Dynamic faces and voices are encountered more often together than in isolation. It is therefore important to determine whether men's preferences observed in experiments that present faces and voices separately are similar to those observed when faces and voices are presented together. Only one other study thus far has investigated human mate preferences with the simultaneous presentation of cross-modal dynamic stimuli. Previously, O'Connor and colleagues presented women raters with brief video clips in which men's voices and faces had been manipulated in masculinity [38]. Findings from this study replicated correlated preferences for male vocal and facial masculinity, but all participants who rated video clips were female. Here, we investigate men's preferences for faces and voices presented simultaneously in video format.

## Methods

### 1.1 Participants

Protocols for this study were approved by the McMaster Research Ethics Board. Informed consent was obtained in writing. Male participants (*N* = 128; mean age ± SD  = 18.31±0.83 years) were recruited from McMaster University and compensated with course credit for participation. All participants were heterosexual as assessed via the Kinsey Scale of Sexual Orientation [39].

### 1.2 Stimuli

Male (*N* = 5; mean age ± SD  = 18.00±0.71 years) and female (*N* = 5; mean age±SD = 17.60±0.55 years) undergraduates were filmed speaking the English word “one”. Despite the fact that stimuli presentation in the current study was brief (approximately 1 s), previous work has demonstrated that 100 ms exposure to a face influences attractiveness judgments to an extent that is indistinguishable from longer exposure times [40]. This method of video stimuli collection and the duration of video presentation have been previously demonstrated to influence vocal and facial masculinity preferences [38].

Recordings were made under standardized lighting conditions in an anechoic sound attenuated booth (Whisper Room SE 2000). Videos were captured with a Panasonic AG-HVX200P video camera with a progressive scan rate of 23.98 frames per second, 24-bit colour depth and a 9×16 aspect ratio. We used this 9×16 aspect ratio instead of the more common 16×9 aspect ratio such that we could maximize the space in each frame that was filled with face. The video camera was white balanced using ExpoDisc. Audio was captured using an external Sennheiser MKH 70 cardioid condenser microphone input to the video camera with a 48 kHz audio frequency sampling rate, and 16-bit amplitude quantization in Adobe On Location CS3 software.

We created our facial stimuli by manipulating the masculinity of each individual's face shape in each frame of the video [41]. Still images were extracted from each uncompressed AVI file, and were converted to uncompressed TIFF format using Adobe Premiere Pro. At no point was pixel dimension altered, nor was any further compression used until the final render. Next, one male and one female prototype were made by averaging together 32 same-sex facial images in colour, shape, and texture [4]. These prototypes served as endpoints when manipulating images in femininity/masculinity. To control for symmetry, which has been found to be associated with masculinity ([42], [43], c.f. [44]), prototypes were made symmetrical by averaging the shape, colour, and texture of each face with its mirror image [4] and did not include the faces of any participants who rated stimuli in this study.

**Figure 1:**
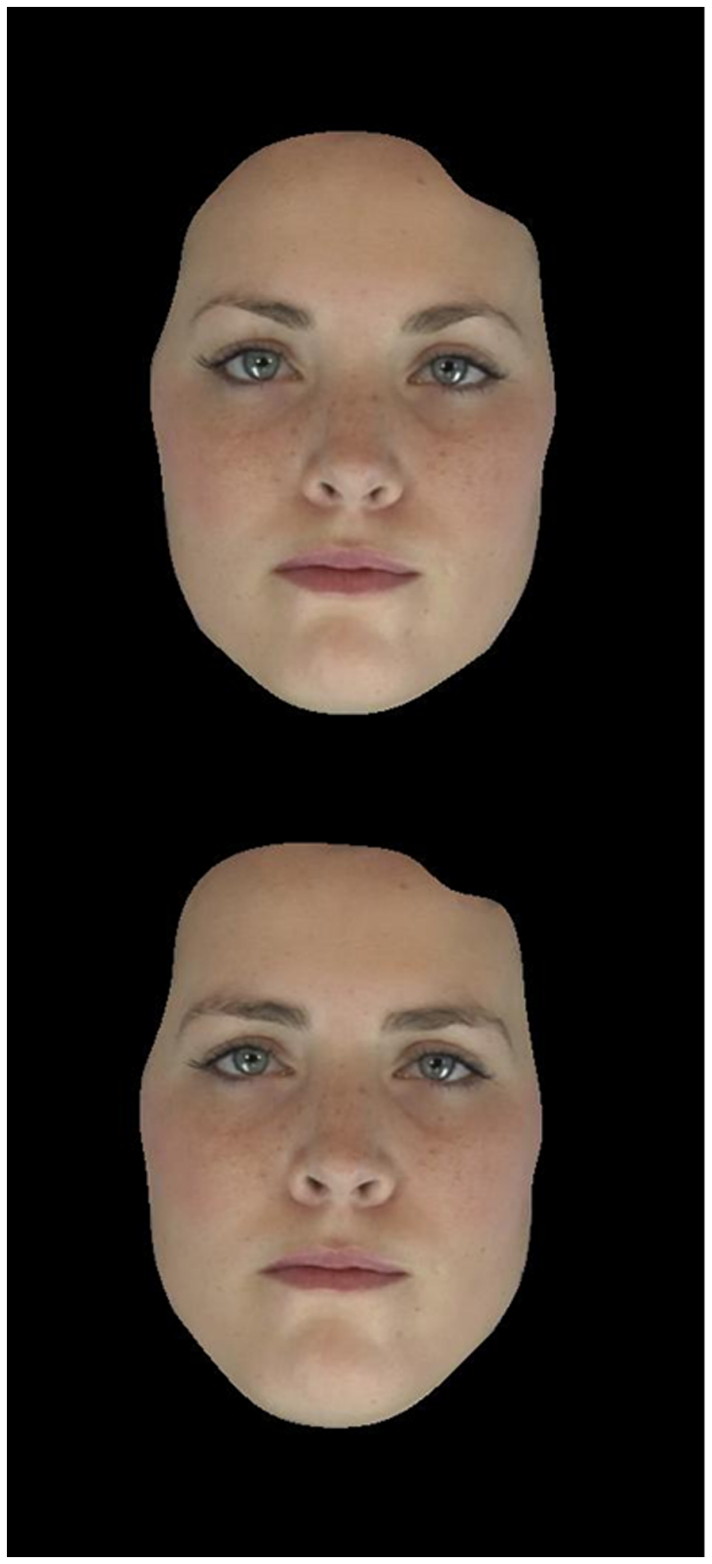
Example of feminized (top) and masculinized (bottom) facial stimuli. *Note.* Frame extracted from video stimulus. Participant provided written informed consent for the publication of this photograph.

We then manipulated facial femininity/masculinity to create a masculinized and feminized version of each frame (for example, see Figure 1) by adding (masculinized) or subtracting (feminized) 50% of the difference in shape between the male and female prototypes. Prototype-based image transformations were carried out using computer graphics software [45]. We standardized inter-pupillary distance and masked the images to reduce cues such as hairstyle, which have been demonstrated to influence masculinity preferences [46].

Audio files were extracted as WAV files using Adobe Premiere Pro. We manipulated voice pitch using the pitch synchronous overlap add method (PSOLA, France Telecom) in Praat software [47] to create a feminized (raised pitch) and masculinized (lowered pitch) version of each audio recording. This method of voice manipulation selectively manipulates fundamental frequency and related harmonics while controlling for other spectrotemporal features of the acoustic signal [48], [49].

Voice pitch was manipulated by raising or lowering voice pitch by ±0.5 equivalent rectangular bandwidths (ERBs) of the baseline frequency. The ERB scale more precisely accounts for the difference between pitch production and pitch perception than do alternative scales [50]. The resulting change in pitch is approximately equivalent to a 25 Hz manipulation, given an average female voice pitch of 225 Hz, and a 20 Hz manipulation given an average male voice pitch of 120 Hz [51]. Post-manipulation voice pitch was within the normal range (female voices: 197–275 Hz; male voices: 111–159 Hz). This level of pitch manipulation has been used in previous research on voice pitch preferences [5], [8].

Masculinized and feminized audio and still images were re-compiled as AVI files in Adobe Premiere Pro. Videos were converted to MPEG-4 container format at an image size of 490×425 pixels, with a 9×16 aspect ratio, 24-bit colour, using the H.264 video codec, with an audio sampling rate of 44.1 kHz, and 16-bit amplitude quantization with the AAC audio codec using QuickTime Pro. This resulted in 4 videos per voice-face masculinity combination (masculine voice and face, masculine voice and feminine face, feminine voice and masculine face, feminine voice and face), with 20 videos per stimuli sex, for a total of 40 videos (stimuli available upon request). This method of stimulus manipulation has been previously demonstrated to influence attractiveness ratings [38].

Male participants viewed and then rated each video (see Videos S1–S4 for an example) for attractiveness on a 7-point scale from 1 (very unattractive) to 7 (very attractive). Participants initiated video playback on a computer monitor (30” Apple Cinema Display, monitor resolution 2560×1080, 24 bit colour). Dynamic video playback duration was approximately one second and videos were visible on-screen until participants entered their attractiveness rating. Participants were allowed unmonitored ad-libitum repetitions of each video. Videos were blocked by stimuli sex. Within each block, videos were fully randomized for order of presentation and played sequentially. The order of blocks was also randomized between participants.

## Results

All analyses were carried out with SPSS 18 (data available upon request), using two-tailed probability estimates (α = 0.05). Inter-rater agreement on video stimuli attractiveness was excellent (>0.9) for both male (Cronbach's alpha  = .953) and female stimuli (Cronbach's alpha  = .945).

**Figure 2:**
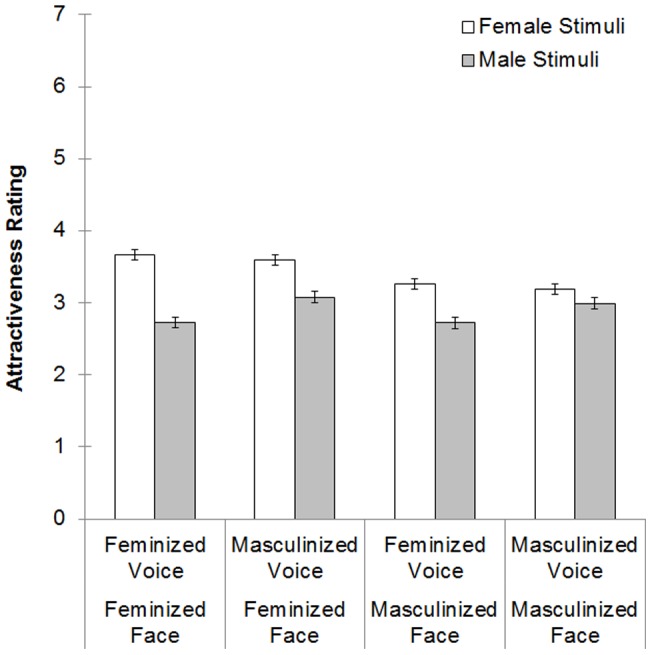
Mean and SEM of attractiveness ratings per stimuli category, per sex of stimuli.

To investigate preferences for vocal and facial masculinity, we averaged the attractiveness ratings of each stimulus within each category to create an attractiveness score per voice-face combination for each participant (see Figure 2). Attractiveness scores were analyzed with a repeated measures ANOVA [within-subject factors: sex of stimuli (female, male), facial masculinity (feminine, masculine), and vocal masculinity (feminine, masculine)]. There was a significant main effect of sex of stimuli (*F*_1, 128_  = 59.57, *P*<.001, η_p_^2^  = .297), where female stimuli (M ± SE  = 3.45±0.066) were given significantly higher attractiveness ratings than were male stimuli (M ± SE  = 2.88±0.075). We observed a significant main effect of facial masculinity (*F*_1, 128_  = 115.15, *P*<.001, η_p_^2^  = .476), where feminized faces (M ± SE  = 3.27±0.061) were rated significantly more attractive than were masculinized faces (M ± SE  = 3.04±0.060). There was also a significant main effect of vocal masculinity (*F*_1, 128_  = 20.76, *P*<.001, η_p_^2^  = .141), where masculinized voices (M ± SE  = 3.21±0.063) were rated as significantly more attractive than were feminized voices (M ± SE  = 3.10±0.059). We also found a significant interaction between facial masculinity and sex of stimuli (*F*_1, 128_  = 56.25, *P*<.001, η_p_^2^  = .307) and between vocal masculinity and sex of stimuli (*F*_1, 128_  = 51.18, *P*<.001, η_p_^2^  = .287). There were no other significant main effects or interactions (all *F*≤1.44, all *P*≥.232).

To investigate the interactions between sex of stimuli and vocal masculinity, and between sex of stimuli and facial masculinity, we added the attractiveness ratings within each level of facial masculinity manipulation (e.g. masculinized face with masculinized voice + masculinized face with feminized voice), and then took the average to create a mean attractiveness rating for masculinized faces and a mean attractiveness rating for feminized faces, for each sex separately. The same was done for each level of vocal masculinity, which resulted in a mean attractiveness rating for masculinized voices, and a mean attractiveness rating for feminized voices, independently for each sex.

A paired *t*-test indicated that both feminized faces (*t*_127_  = 9.04, *P*<.001) and masculinized faces (*t*_127_  = 4.85, *P*<.001) were rated as more attractive when the stimuli were female than when they were male. Attractiveness ratings of feminized women's faces were higher than were attractiveness ratings of masculinized women's faces (*t*_127_  = 10.75, *P*<.001). Attractiveness ratings of feminized men's faces were higher than attractiveness ratings of masculinized men's faces (*t*_127_  = 2.18, *P*  = .003). A paired *t*-test also indicated that both feminized voices (*t*_127_  = 9.29, *P*<.001) and masculinized voices (*t*_127_  = 4.53, *P*<.001) were rated as more attractive when stimuli were female than when they were male. Vocal femininity was rated as significantly more attractive than vocal masculinity when stimuli were female (*t*_127_  = 2.67, *P* = .009). When stimuli were male, vocal masculinity was rated as significantly more attractive than vocal femininity (*t*_127_  = −6.83, *P*<.001).

In order to investigate the relationship between vocal and facial masculinity preferences, we created a vocal masculinity preference score and a facial masculinity preference score, for each sex separately. Vocal masculinity preferences were calculated by subtracting attractiveness ratings of feminized voices from attractiveness ratings of masculinized voices, for each level of facial masculinity/femininity manipulation. This resulted in two scores: preference for masculinized male voices paired with feminized faces, and preference for masculinized male voices paired with masculinized faces. We then averaged these two preference scores. This resulted in a general vocal masculinity preference score, where higher numbers reflect higher average attractiveness ratings independent of the masculinity of the other modality. The same method was used to calculate general facial masculinity preference scores. General facial and vocal masculinity preference scores for female stimuli were reverse coded to create general facial and vocal femininity preferences. A Pearson correlation indicated that men who preferred feminized female voices also preferred feminized female faces (*r* = .194, *N* = 128, *P* = .029), but the relationship between men's preferences for male vocal masculinity and male facial masculinity was not significant (*r* = −.101, *N* = 128, *P* = .255).

## Discussion

In the present study, men rated the attractiveness of brief video recordings of men and women whose faces and voices had been manipulated to be relatively more feminine or more masculine. We found that men rated feminine women's faces and voices significantly more attractive than they rated masculine women's faces and voices. Men rated feminine men's faces as more attractive than they rated masculine men's faces, and masculine men's voices as more attractive than they rated feminine men's voices. Men's preferences for vocal and facial femininity were significantly and positively correlated when stimuli were female, but not when stimuli were male.

We found significant preferences among men for feminine women's faces and voices. Men's preferences for such feminine female traits may be due to the relationships among youth, fertility, and the expression of estrogen and testosterone. Indeed, women possessing traits indicative of higher levels of estrogen and lower levels of testosterone, such as feminine faces and voices, may be healthier, younger, and more fertile mates [13], [14], [33]. Moreover, men may also prefer higher pitched women's voices over lower pitched women's voices because raised voice pitch may be indicative of female attraction to a potential mate [52]. Many other studies utilizing either soundless still images of women's faces [4], [5], [53] or audio-only recordings of women's voices [1], [8], [12], [54] have also found significant preferences for female femininity over masculinity. Therefore, the observation of men's preferences for femininity in women's faces and voices is not dependent upon the manner of stimuli presentation.

We found that men who preferred feminized women's faces also preferred feminized women's voices. This supports prior findings that men's ratings of women's vocal and facial femininity are positively correlated, specifically when men were rating women as long-term mates [5]. While voices and faces were presented separately in that study, here we presented men with female voices and faces simultaneously [5]. Therefore, men's preferences for vocal and facial female femininity observed in studies where voices and faces are presented separately are similar to preferences observed when voices and faces are presented simultaneously. Here, men's preferences for facial and vocal femininity were influenced to a similar degree by the presence of either vocal or facial femininity, respectively. This pattern of results is similar to those observed when women rated the attractiveness of the men in videos whose voices and faces were manipulated to be more or less masculine [38]. This finding complements a growing body of literature demonstrating correlated preferences among men for cross-modal cues to women's mate quality, such as vocal and facial femininity [5] and putative female pheromones and facial femininity [55]. Correlated preferences for cross-modal cues to underlying mate quality may be adaptive if it aids in the selection of higher quality mates [3].

When men were presented with videos of other men manipulated to possess more or less facial and vocal masculinity, men rated feminized men's faces as more attractive than masculinized men's faces. Other studies of heterosexual men's preferences for other men's faces have observed preferences for both masculinity [6] and femininity [4], [56]. Similarly, we found that men rated masculinized male voices as more attractive than feminized male voices. Other work has demonstrated that men prefer masculine male voices [54], [57], or that men's preferences for other men's voices do not differ from chance [11], [56]. While the nature of same-sex attractiveness ratings among heterosexual individuals has yet to be determined, current evidence supports that same-sex attractiveness ratings are, to some extent, associated with perceptions of intrasexual competition. Specifically, heterosexual men who rate masculinized male faces and voices as more attractive than feminized male faces and voices report more jealousy in response to hypothetical intrasexual competitors with masculinized, rather than feminized, faces and voices [56]. However, the influence of masculinity manipulations on heterosexual men's same-sex attractiveness ratings are not entirely accounted for by perceptions of other men's dominance [54]. Future studies may elucidate the nature of heterosexual men's same-sex attractiveness ratings.

In the current study we did not detect a significant relationship between men's ratings of masculinized male faces and voices. In contrast, other research has observed significant positive correlations between men's preferences for masculine male voices and faces when stimuli were presented separately, but sexual orientation was not reported in that study [57]. Our finding that men's preferences for vocal and facial femininity were correlated among female but not male stimuli complements other work that finds that variation in both heterosexual and homosexual men's preferences for facial masculinity is specific to the sex with whom those men prefer to mate [6]. Collectively, this research suggests that variation in preferences facilitates men's mate search behaviour, whether it is for male or female mates. Furthermore, by demonstrating that men's preferences for feminized/masculinized voices and faces in videos are different for female and male stimuli, we can be sure that judgements reported here are not due to a general response bias towards masculine or feminine stimuli.

We did not find a significant interaction between vocal and facial masculinity preferences for either male or female stimuli. We did find that when stimuli were female, stimuli manipulated to be more feminine in either modality were rated as significantly more attractive than when they were manipulated to be more masculine. When stimuli were male, the pairing of a masculinized voice (or face) increased attractiveness ratings regardless of whether faces (or voices) where feminine or masculine. Other research has found that preferences for other masculine characteristics do interact. For example, women's preferences for lower pitched men's voices relative to higher pitched men's voices were greater when the apparent vocal tract length was manipulated to be larger rather than smaller [58]. Future studies may investigate whether attractiveness judgements are influenced by interactions between other cues to underlying mate quality.

One potential explanation for the lack of an interaction between vocal and facial masculinity on attractiveness ratings concerns the magnitude of voice pitch versus facial masculinity manipulations. Here, voice pitch was manipulated to be higher or lower by 0.5 equivalent rectangular bandwidths (ERBs) of the baseline frequency, resulting in a manipulation of approximately 20–25 Hz. Faces were manipulated relative to baseline to be either 50% more masculine or 50% more feminine. However, it is unclear whether the degree of voice pitch manipulation was perceptually equivalent to the degree of facial masculinity manipulation. More or less exaggerated manipulations of masculinity may result in a significant interaction between vocal and facial masculinity on men's perceptions of attractiveness, and is an avenue for future research.

Participants in the present study initiated video playback with a mouse click. The first and last still frames of the video were visible onscreen before and after (respectively) participants played the video. In effect, participants were viewing still images at the beginning and end of the video. Hence, it is possible that these still images influenced participant's attractiveness ratings of the video. However, if participants' attractiveness ratings were based solely upon these still images, we would not have found a significant effect of voice pitch manipulation on attractiveness ratings. Indeed, we observed that vocal femininity positively influenced attractiveness ratings when stimuli were female whereas vocal masculinity positively influenced attractiveness ratings when stimuli were male.

In summary, we presented men with short video clips of women and men whose faces and voices had been manipulated to be more or less feminine or masculine. We found that men preferred feminine women's voices and faces, feminine men's faces, and masculine men's voices. Men's attractiveness ratings of feminine faces did not depend on the level of vocal masculinity, and men's attractiveness ratings of feminine voices did not depend on the level of facial femininity present in the video. Men's preferences for feminine female voices and faces were significantly positively correlated, but men's preferences for masculine male faces and voices were not. These findings demonstrate that preferences observed in studies of the influence of facial and vocal femininity on men's mate preferences are independent of whether stimuli are presented separately or simultaneously. Cross-modal preferences for vocal and facial femininity, such as those observed here, may facilitate male mate search behaviour and may serve as a system to verify honest cueing of underlying female condition.

## Supporting Information

Video S1 **Example of video stimuli with masculinized face and masculinized voice.**
(MOV)

Video S2 **Example of video stimuli with feminized face and feminized voice.**
(MOV)

Video S3 **Example of video stimuli with feminized face and masculinized voice.**
(MOV)

Video S4 **Example of video stimuli with masculinized face and feminized voice.**
(MOV)
